# Constitutive Expresser of Pathogenesis Related Genes 1 Is Required for Pavement Cell Morphogenesis in *Arabidopsis*

**DOI:** 10.1371/journal.pone.0133249

**Published:** 2015-07-20

**Authors:** Bing Han, Liang Chen, Jing Wang, Zhongliang Wu, Longfeng Yan, Suiwen Hou

**Affiliations:** Ministry of Education Key Laboratory of Cell Activities and Stress Adaptations, School of Life Sciences, Lanzhou University, Lanzhou, 730000, People’s Republic of China; Northeast Forestry University, CHINA

## Abstract

For over 50 years, researchers have focused on the mechanisms underlying the important roles of the cytoskeleton in controlling the cell growth direction and cell expansion. In our study, we performed ethyl methane sulfonate mutagenesis on Col-0 background and identified two new *CONSTITUTIVE EXPRESSER OF PATHOGENESIS RELATED GENES 1 (CPR1)* alleles with pavement cell (PC) morphogenetic defects. Morphological characterizations showed that polar growth initiation and expansion of PCs are seriously suppressed in *cpr1*. Closer cytoskeleton investigation showed that the directional arrangement of microtubules (MTs) during PC development is defective and the cortical fine actin filaments cannot be aggregated effectively to form actin cable networks in *cpr1* mutants. These results suggest that the abnormal PC morphogenesis in *cpr1* is accompanying with the aberrant arrangement of cytoskeleton. Site-directed mutagenesis and knockout within the F-box-associated (FBA) domain, which is reported to be a motif for recognizing particular substrates of CPR1, proved that the FBA domain is indispensable for normal CPR1 regulation of the PC morphogenesis. Further genetic analysis indicated that the defects on PC morphogenesis of *cpr1* depend on two lipase-like proteins, ENHANCED DISEASE SUSCEPTIBILITY 1 and PHYTOALEXIN DEFICIENT 4. Our results provide further insights into the relationship between the cytoskeleton and PC morphogenesis, and suggest that the cytoskeleton-mediated PC morphogenesis control might be tightly linked to plant defense responses.

## Introduction

Cell shape formation and regulation are crucial for cell function, tissue and organ development, and morphogenesis in plant [1–3]. The control mechanisms of the cell shape have attracted great interest. Leaf epidermal pavement cell (PC) with a multi-polar growth pattern exhibits complex cell shape and serves as an exciting model to investigate the mechanisms of cell shape formation [4–7]. By studying mutants with impaired PC morphogenesis, researchers have revealed that the cytoskeleton is central to PC shape formation, just like in cylindrical hypocotyl cells [6,8].

The distribution and arrangement of the cytoskeleton, including microtubules (MTs) and microfilaments (MFs), are crucial for PC morphogenesis [9,10]. Well-ordered transverse cortical MTs promote cell elongation and restrict cell expansion on the direction of their dominant orientation [7]. In PC, parallel microtubule bundles arrange transversely in the neck regions of PC and restrict the local outgrowth [11,12]. In contrast, cortical MFs localize to the sites lacking well-ordered cortical MTs, and promote local growth, leading to the lobe initiation and outgrowth [9,13]. In addition, the coordination between the microtubule and microfilament cytoskeleton plays an important role in complex cell shape formation. Actin cables arrange along the growth axis and dynamic cortical fine F-actin localizes to the tip leading to cell growth. During this process, the regular microtubule cytoskeleton is required [14,15]. In return, the microtubule re-assembly also requires the actin cytoskeleton [16].

Cell morphogenesis and polarity maintenance in higher plant are dependent on the inter-cellular and intra-cellular communication [15]. *Arabidopsis thaliana* Rho-like guanosine triphosphatase (ROP-GTPase) is a plant-specific member of the Rho GTPase family, which plays an important role in cytoskeleton morphogenesis in PC [8,9,17]. Two counteracted ROP-mediated signal pathways regulate the polarity outgrowth and cell expansion in PCs [11]. ROP2 and ROP4, two functionally redundant members, which are locally activated at the lobe-forming site, promote lobe outgrowth by activating ROP-INTERACTIVE CRIB MOTIF-CONTAINING PROTEIN 4 (RIC4)-mediated assembly of fine cortical MFs and suppress well-ordered cortical MT arrays by inactivating another effector, RIC1 [11,18]. ROP6-activated RIC1-microtubule pathway promotes neck formation through the activation of KATANIN 1-mediated microtubule severing and suppresses ROP2 activation (Lin et al. 2013).

An F-box protein, CONSTITUTIVE EXPRESSER OF PATHOGENESIS RELATED GENES 1 (CPR1), is expressed in most tissues, such as seedlings, root, stem, rosette, inflorescence and silique, and is localized in the cytoplasm and nucleus [19,20]. During plant defense responses, CPR1 interacts with a TIR-NBS-LRR R protein, SUPPRESSOR OF NPR1-1 (SNC1), negatively regulating the accumulation of SNC1 through the 26S proteasome in *Arabidopsis* [21,22]. Two lipase-like proteins, ENHANCED DISEASE SUSCEPTIBILITY 1 (EDS1) and PHYTOALEXIN DEFICIENT 4 (PAD4), positively regulate TIR-NB-LRR type of R gene-mediated defense responses [23,24]. Recent reports demonstrate that functional EDS1, PAD4, and SNC1 are required for the *cpr1*-conferred defect in defense responses and this signaling pathway is temperature-sensitive [20,25]. In our study, we screened and identified two *cpr1* alleles with PC morphogenesis defects. The arrangement of MTs and MFs were distinctly disrupted in *cpr1* mutants. Genetic analysis showed that CPR1 is required for proper PC shape formation independent of ROP-GTPase signaling pathway. However, loss-of-function *PAD4* and *EDS1* can completely rescue the defects of PC morphogenesis in *cpr1* mutants. Our results suggest that CPR1-regulated cytoskeleton arrangement controls PC morphogenesis, which is involved in the natural plant defense responses, and that the FBA domain is indispensable for normal CPR1 function.

## Materials and Methods

### Plant materials and growth conditions

*Arabidopsis thaliana* Columbia-0 (Col-0) was used as the wild type (WT). Plants were sown and grown in a controlled environment under a 16 h light/8 h dark cycle at 22°C or 28°C. The mutants and transgenic plants used in the present study were as follows: *cpr1* (T-DNA line, SALK_045148), GFP-tagged α-tubulin TUA6 (GFP-TUA6 transgenic line, CS6551), GFP-tagged to the second actin-binding domain of *Arabidopsis* fimbrin 1 (GFP-FABD2, transgenic line), *rop2* (T-DNA line, CS855973), *rop6* (T-DNA line, SALK_091737), *eds1* (T-DNA line, SALK_057149), and *pad1* (T-DNA line, CS859090). The double mutants *eds1 cpr1-j594*, *eds1 cpr1-j2928*, *pad4 cpr1-j594*, *pad4 cpr1-j2928*, *rop2 cpr1-j594*, *rop2 cpr1-j2928*, *rop6 cpr1-j594*, *rop6 cpr1-j2928* and GFP-tagged lines *cpr1-j594*:TUA6-GFP, *cpr1-j2928*:TUA6-GFP, *cpr1-j594*:FABD2-GFP, and *cpr1-j2928*:FABD2-GFP were also used.

### Genetic mapping of the mutant gene

*cpr1-j594* and *cpr1-j2928* (Columbia background) were backcrossed to L*er Arabidopsis* plants to establish a mapping population. The F2 progenies with mutant phenotype were used for map-based cloning and DNA from homozygote individuals was isolated for molecular mapping. A set of simple sequence length polymorphism (SSLP) and cleaved amplified polymorphic sequence (CAPS) markers were used to map the mutant gene. The markers were obtained from the *Arabidopsis* mapping platform (AMP) [26].

### PC morphometric analysis

PCs from apical sectors of mature leaves were used for quantitative analysis of PC area, circularity, lobe length, and lobe number [11, 27]. The images were collected using a HITACHI S-3400N scanning electron microscope. The equation used for circularity calculation is as follows: 4π area/perimeter^2^ [27]. For each genotype, approximately 100 highly expanded cells, from at least ten independent leaves were measured.

### MT and MF visualization using confocal microscopy

Both *cpr1-j594* and *cpr1-j2928* were crossed to transgenic plants expressing GFP-TUA6 and GFP-FABD2, respectively. F2 plants with steady green fluorescent protein were used for MT and MF analysis. Images were collected using a confocal microscope (LeicaTCS SP8).

### Developmental characterization of PCs

Approximately 0.5 cm long wild-type abaxial leaf surface was observed by scanning electron microscope (SEM). Based on the positions of cells along the long axis of the leaf, the development of PCs was divided into three stages. Stage I, cells were localized in the base of the leaf. Stage II, cells were localized around the midpoint of the leaf. Stage III, cells were localized in the tip of the leaf. The different developmental stages of PCs were identified in *cpr1-j594* and *cpr1-j2928* plants based on their leaf positions with respect to those in wild-type leaves [9]. The images were collected using a HITACHI S-3400N SEM.

### Plasmid construction and generation of transgenic plants

Gateway technology was employed for most genetic manipulations. For the complementation test, 3.5 kb of the genomic sequence including the full *CPR1* genomic sequence and its 1.9 kb upstream sequence was amplified, and cloned into the pBIB-BASTA-GWR-GFP vector by *in vitro* DNA recombination (see primers in supplementary material S1 Table). For the overexpression construct, the CDS sequence of *CPR1*, *CPR1I247V*, and *CPR1∆FBA* were amplified, and introduced into the destination vector pB35GWF (see primers in supplementary material S1 Table). All the cloned sequences were confirmed by sequencing analysis.

## Results

### *j594* and *j2928* show severe PC morphogenetic defects

**Fig 1:**
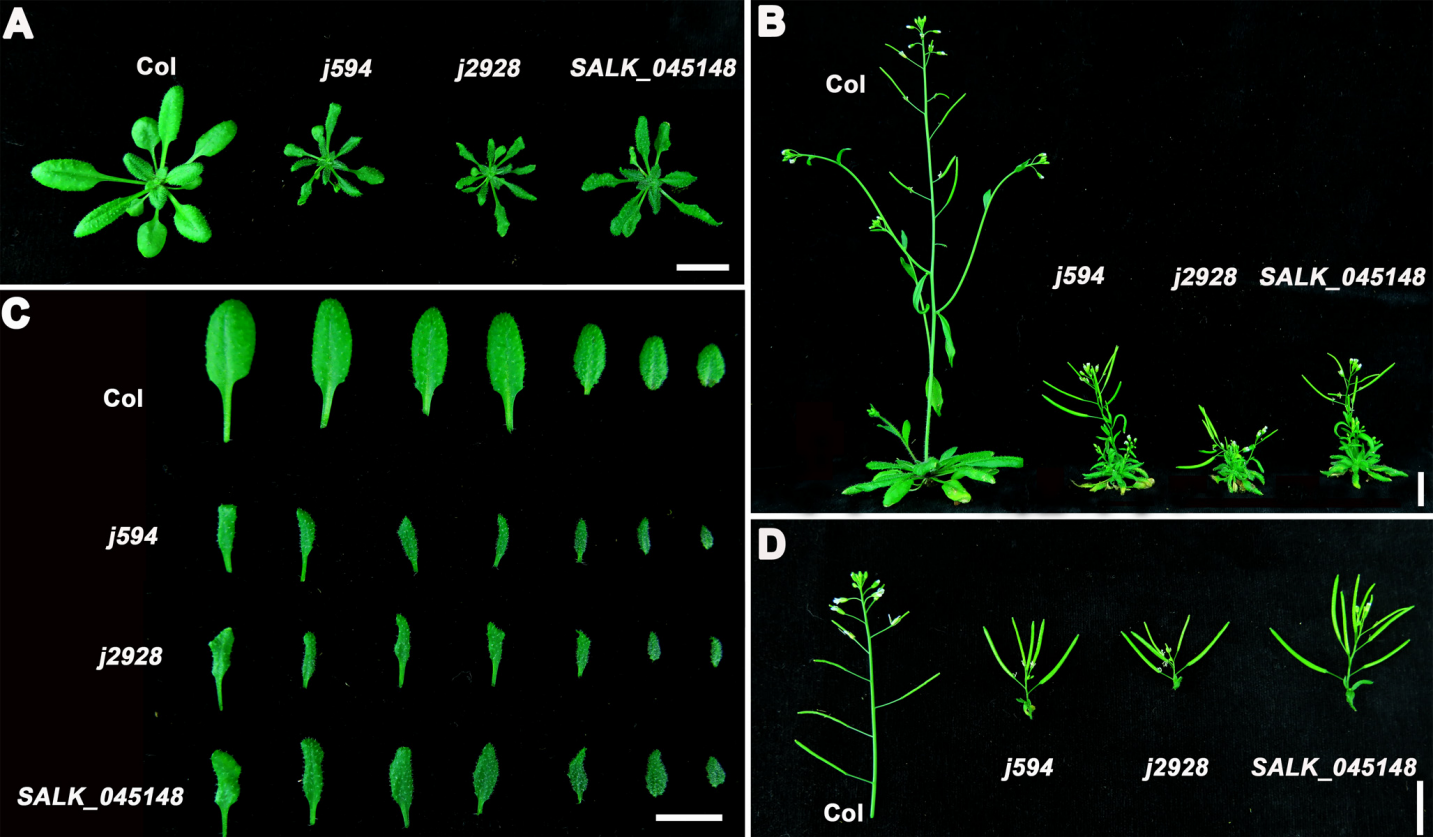
Phenotypic analysis of Col, *j594*, *j2928* and SALK_045148 plants. **(A)** Two-week-old seedlings of Col, *j594*, *j2928* and SALK_045148. **(B)** Five-week-old seedlings of Col, *j594*, *j2928* and SALK_045148. **(C)** Photographs of representative leaf series of Col, *j594*, *j2928* and SALK_045148 (the fifth true leaves on the left and the youngest leaves on the right). **(D)** Siliques of Col, *j594*, *j2928* and SALK_045148. Bars = 1 cm.

**Fig 2:**
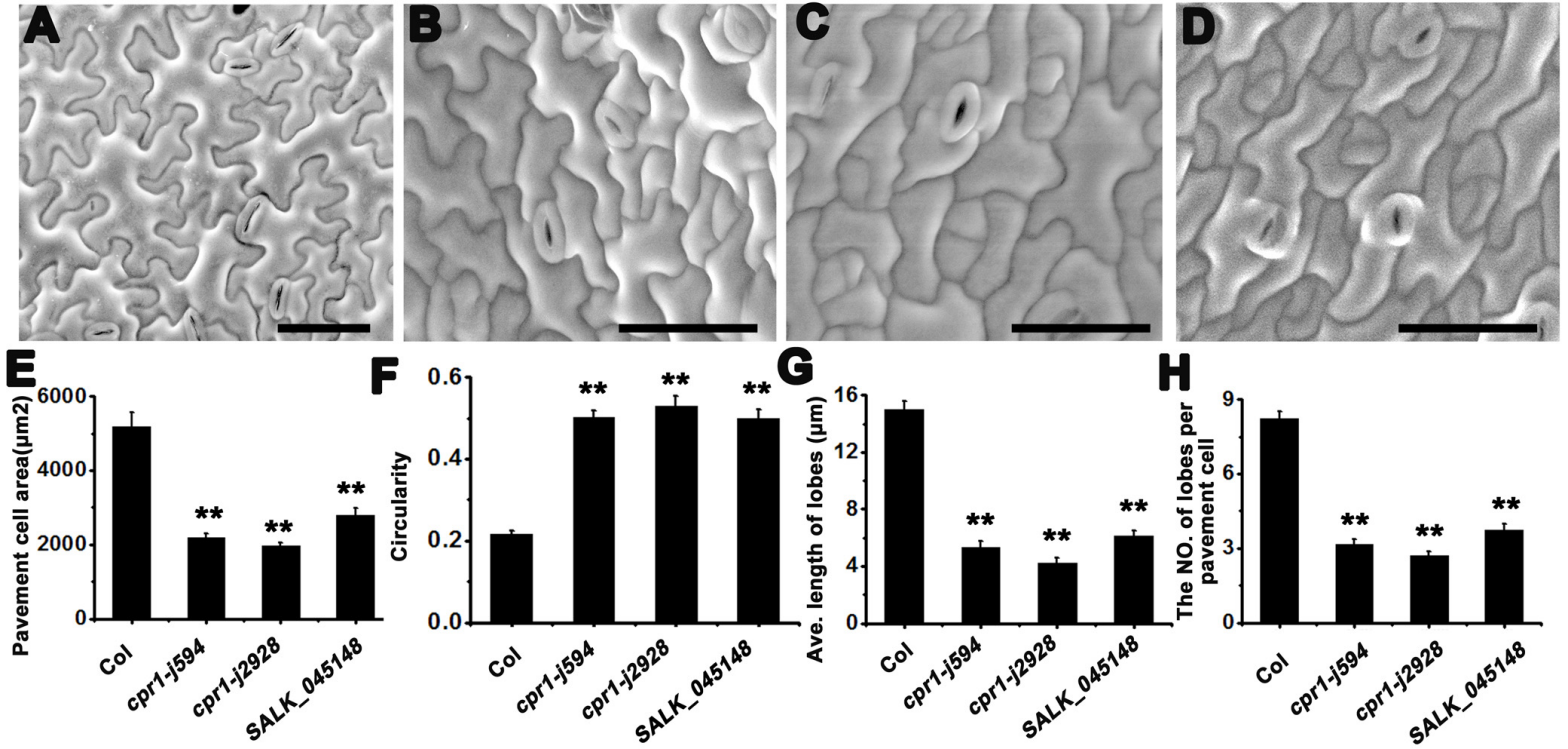
PC phenotype in Col, *j594*, *j2928* and SALK_045148. **(A–D)** Mature PC shapes of Col (**A**), *j594* (**B**), *j2928* (**C**), and SALK_045148 (**D**). Bars = 50 μm. **(E–H)** Quantitative analyses of the PC area (**E**), circularity (**F**), lobe length (**G**), and lobe number (**H**) in Col, *j594*, *j2928* and SALK_045148. Error bars indicate s.e.m.; **, P<0.01 by Student’s test.

To identify more components regulating PC morphogenesis, we performed an ethyl methane sulfonate mutagenesis in Col-0 background seeds and screened more than 3000 independent lines. Two mutants, *j594* and *j2928*, were identified. Compared with wild-type plants, *j594* and *j2928* developed petite seedlings (Fig 1A), narrower leaves (Fig 1C), and shorter shoots (Fig 1B) and silique clusters (Fig 1D). Closer scrutiny by SEM showed abnormal PC shape in *j594* and *j2928* (Fig 2A–2C). Quantitative PC analysis of WT, *j594* and *j2928* using the geometric analysis method [12,27,28] showed that PCs in *j594* and *j2928* were smaller and exhibited less and shorter lobes (Fig 2E, 2G and 2H). Circularity has been reported as a quantitative descriptor of PC shape complexity [27], thus the circularity of PC in WT, *j594* and *j2928* was assayed. The results showed that PCs of *j594* and *j2928* have larger values of circularity compared with wild type (Fig 2F). These results implied that the PCs in *j594* and *j2928* exhibit severe morphogenetic defects.

### Lobe initiation and outgrowth are both inhibited during PC development in *j594* and *j2928*

**Fig 3:**
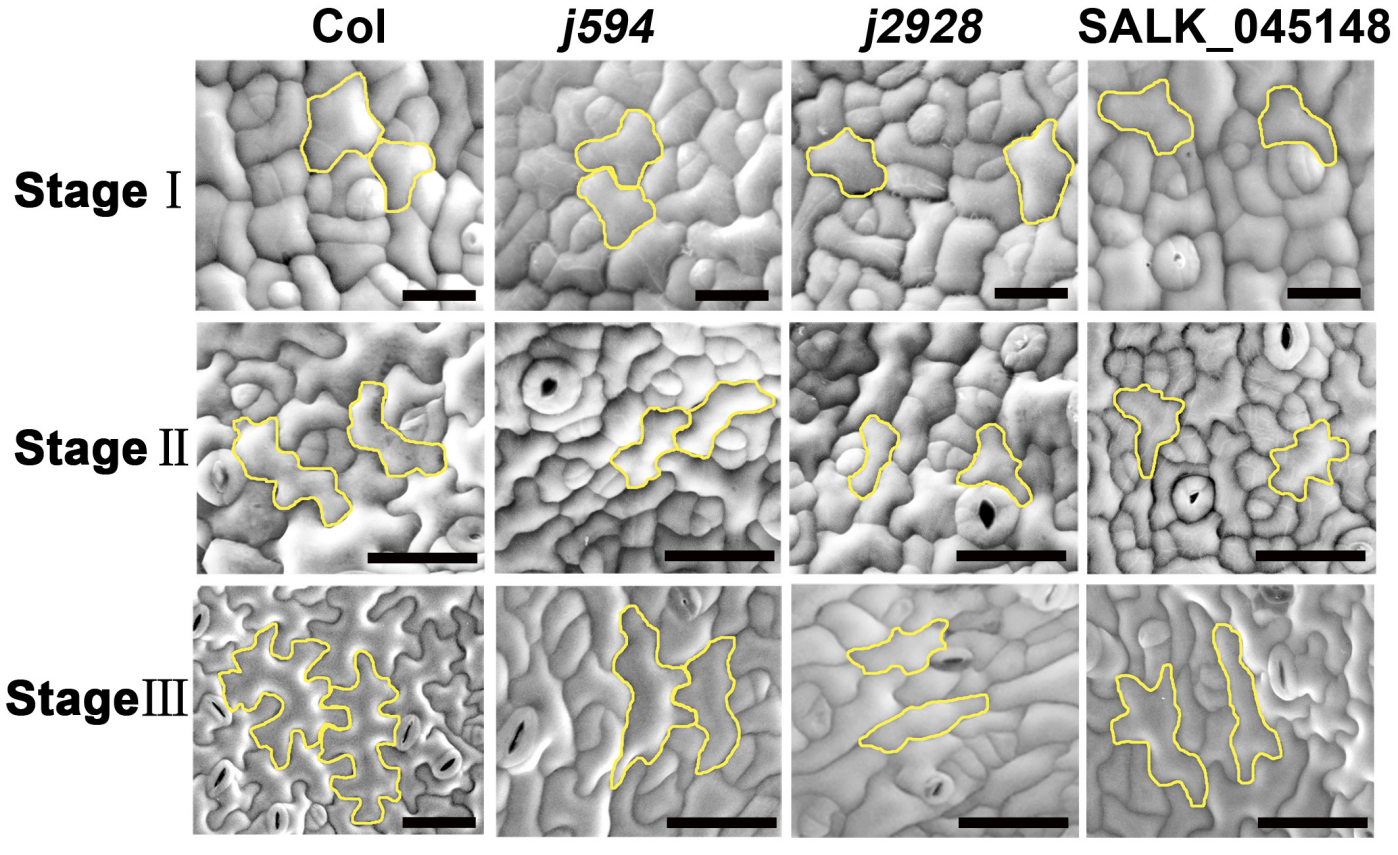
SEM images of PCs at different developmental stages in Col, *j594*, *j2928* and SALK_045148. The top panels, middle panels and bottom panels showed PCs of Col, *j594*, *j2928* and SALK_045148 in stage I, stage II and stage III respectively. The cells with yellow stroke color represented cell shape in each PC developmental stage of Col, *j594*, *j2928* and SALK_045148. Bars = 50 μm.

Previous reports have divided PC development into three stages [9]. To investigate the mechanisms of cell shape formation in *j594* and *j2928* mutants, we recorded PC shape during the different developmental stages in each genotype. During Stage I, *j594* and *j2928* PCs formed slightly elongated polygons as observed in wild-type cells (Fig 3, top panels). During Stage II, PCs initiated multiple outgrowths that resulted in multiple shallow lobes in the wild-type cells, whereas they hardly initiated new lobes in *j594* and *j2928* cells (Fig 3, middle panels, S2 Fig). During Stage III, the multiple shallow lobes fully extended formed a complex shape in the wild-type cells. In contrast, in *j594* and *j2928* PCs, few newly formed polar growth sites were observed and polar extension in this direction was also greatly inhibited (Fig 3, bottom panels). All these results indicated that lobe initiation and outgrowth are both inhibited during PC development in *j594* and *j2928*.

### *j594* and *j2928* are two new alleles of *CPR1*

**Fig 4:**
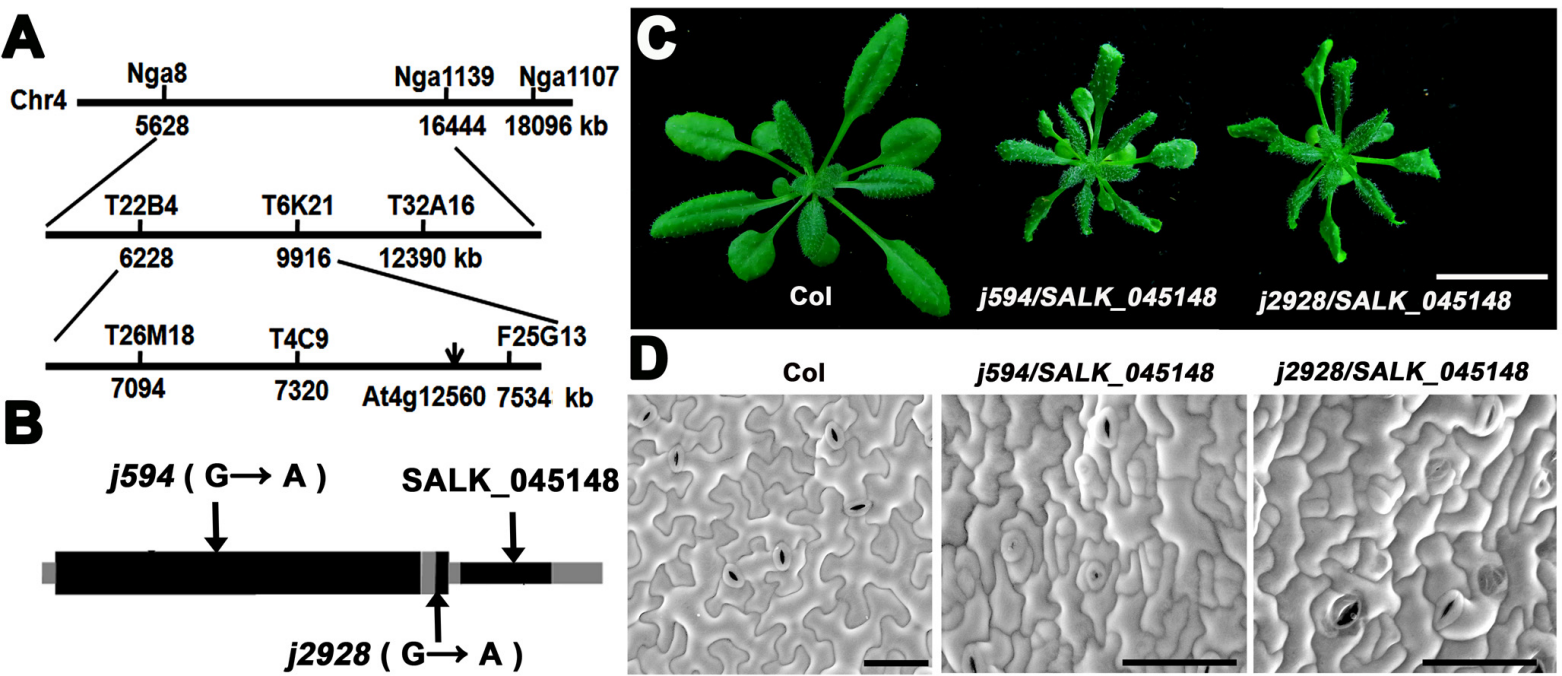
*j594* and *j2928* are two new alleles of *CPR1*. **(A)** Genetic map of the region carrying *CPR1* gene. **(B)** The gene structures of *CPR1* (*At4g12560*.*2*). Black boxes, exons; gray boxes, the untranslated regions (UTRs); gray line, intron. Arrows indicate the positions of the mutated nucleotide and the T-DNA insertion locus. **(C)** Two-week-old seedlings of Col, *j594/*SALK_045148, and *j2928/*SALK_045148. Bar = 1 cm. **(D)** Mature PC shapes of Col, *j594/*SALK_045148, and *j2928/*SALK_045148. Bars = 50 μm.

**Fig 5:**
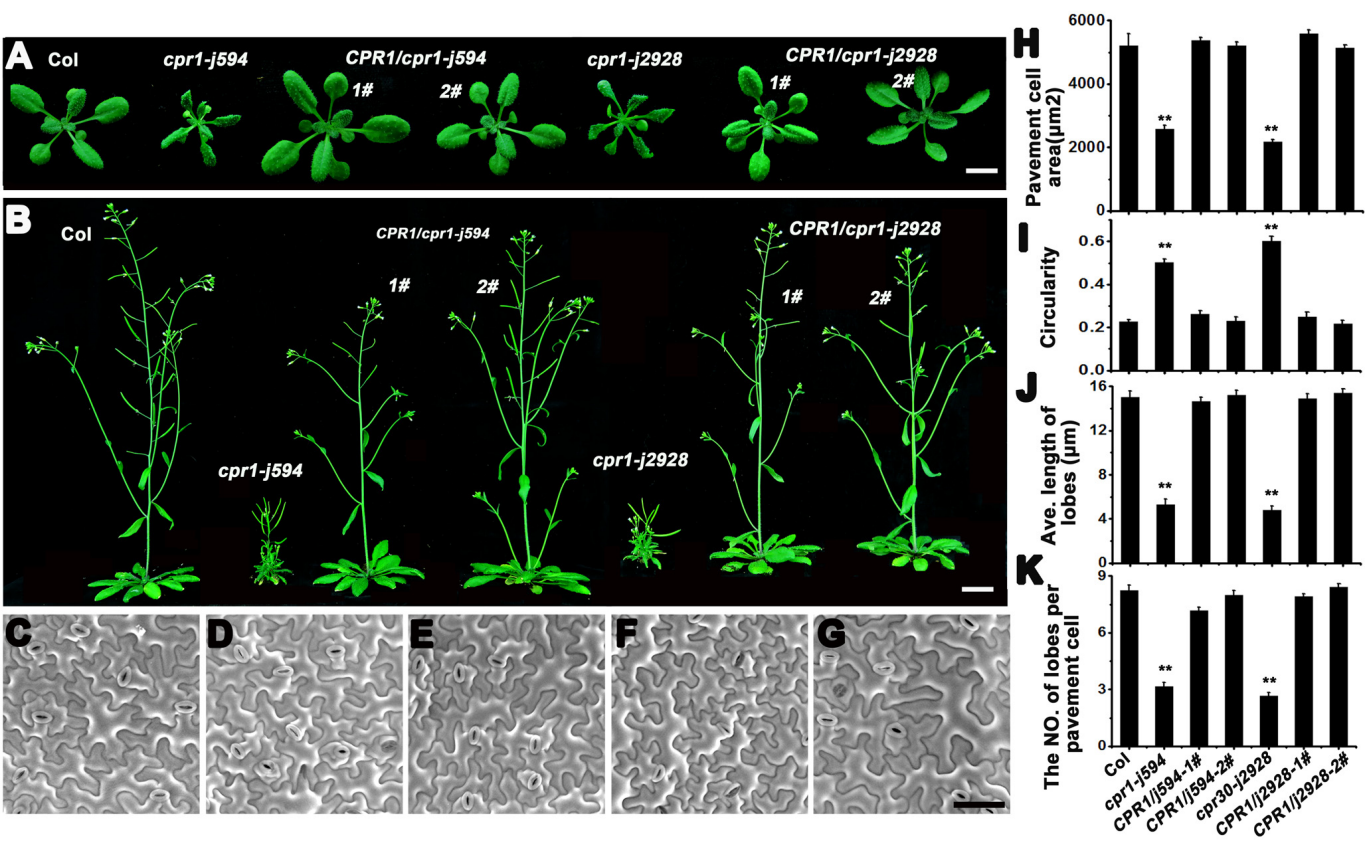
*CPR1* rescues the phenotypes of both *cpr1*-*j594* and cpr1-j2928. **(A)** Two-week-old seedlings of wild-type, *j594*, two *CPR1/cpr1-j594* lines, *j2928*, and two *CPR1/cpr1-j2928* lines. Bar = 1 cm. **(B)** Five-week-old seedlings of wild-type, *j594*, two *CPR1/cpr1-j594* lines, *j2928*, and two *CPR1/cpr1-j2928* lines. Bar = 1 cm. (**C–G)** Mature PC shapes of Col (**C**), *CPR1/cpr1-j594-1#* (**D**), *CPR1/cpr1-j594-2#* (**E**)**,** *CPR1/cpr1-j2928–1#* (**F**), and *CPR1/cpr1-j2928–2#* (**G**). Bar = 50 μm. **(H–K)** Quantitative analysis of the PC in the complementation test. PC area (**H**), circularity (**I**), lobe length (**J**), and lobe number (**K**)**.** Error bars indicate s.e.m.; **, P<0.01 by Student’s test.

To identify the map position of the molecular lesions in these two mutants, map-based cloning was performed. After fine mapping, both of the mutations were located in a 90 kb region on chromosome 4 (Fig 4A) and all the genes in this region were sequenced. Interestingly, we found that both of the mutants have a point mutation in the same gene *At4g12560*, which encodes the F-box protein CPR1 [20]. In *j594*, a G to A mutation at the nucleotide position 181 converted a glycine to an arginine. In *j2928*, a mutation located in the last base of its only intron, lead to the abnormal splice of *CPR1* (Fig 4B). To determine whether *j594* and *j2928* were loss-of-function mutants of *CPR1*, a line with a T-DNA insertion in the 3’-untranslated region of *CPR1* (SALK_045148) was obtained and characterized. The SALK_045148 line exhibited similar phenotypes to *j594* and *j2928* (Fig 1 and Fig 2D). When *j594* and *j2928* were backcross to Col-0, their F1 progenies showed no obvious phenotype (data not shown), indicating that *j594* and *j2928* are recessive mutations of a single gene. They were also crossed to SALK_045148 plants and the F1 progenies from both crosses showed mutant phenotypes (Fig 4C and 4D). These results supported that *j594* and *j2928* are allelic to *CPR1*. To further confirm this result, the native promoter of *CPR1* fused to its full length gene was transformed into *j594* and *j2928*, and all the transgenic lines rescued plant and PC morphogenetic mutant phenotypes of *j594* and *j2928* (Fig 5). Based on these results, we concluded that the phenotype of *j594* and *j2928* is due to the loss function of *CPR1*. Therefore, we renamed them *cpr1-j594* and *cpr1-j2928*, respectively.

### The FBA domain is indispensable for normal CPR1 function

**Fig 6:**
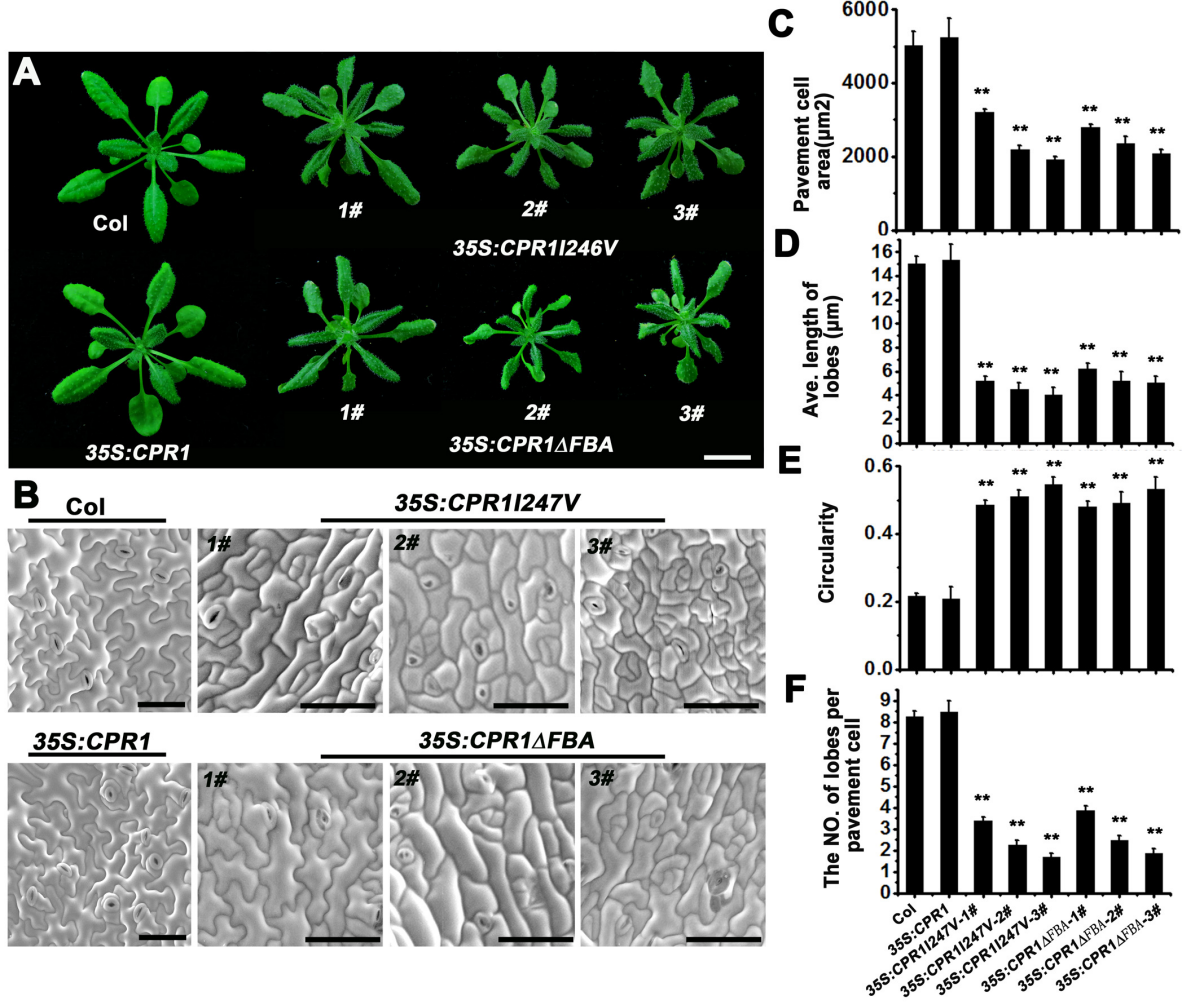
Phenotypic analysis in Col, *35S*::*CPR1*, *35S*::*CPR1I247V* and *35S*::*CPR1∆FBA* transgenic plants. **(A)** Two-week-old seedlings of Col, *35S*::*CPR1*, three *35S*::*CPR1I247V* transgenic lines and three *35S*::*CPR1∆FBA* transgenic lines. Bar = 1 cm. **(B)** Mature PC shape of Col, *35S*::*CPR1*, three *35S*::*CPR1I247V* transgenic lines and three *35S*::*CPR1∆FBA* transgenic lines. Bars = 50 μm. **(C–F)** Quantitative analysis of the PC in the Col, *35S*::*CPR1*, three *35S*::*CPR1I247V* transgenic lines and three *35S*::*CPR1∆FBA* transgenic lines. PC area (**C**), lobe length (**D**), circularity (**E**), and lobe number (**F**)**.** Error bars indicate s.e.m.; **, P<0.01 by Student’s test.

To investigate the function of the FBA domain in CPR1, two *CPR1* overexpression constructs with mutations in the FBA domain were used. The first construct had a point mutation (*35S*::*CPR1I247V*) and the second lacked the FBA domain (*35S*::*CPR1ΔFBA*). Both constructs were expressed under a cauliflower mosaic virus 35S (CaMV35S) promoter in Col-0 background. Transgenic lines of both *35S*::*CPR1I247V* and *35S*::*CPR1ΔFBA* exhibited similar phenotypes to those observed in *cpr1-j594* and *cpr1-j2928* (Fig 6A and 6B). Quantitative PC shape analysis showed that PCs in *35S*::*CPR1I247V* and *35S*::*CPR1ΔFBA* lines are small, have short and reduced lobes, and large values of circularity (Fig 6C–6F). However, plants overexpressing the normal *CPR1* exhibited phenotypes similar to those observed in wild-type plants and PCs (Fig 6A and 6B). Meanwhile, expression of exogenous *CPR1* did not affect the transcription level of endogenous *CPR1* (S1 Fig). Thus, we concluded that *35S*::*CPR1I247V* and *35S*::*CPR1ΔFBA* could imitate the phenotypes of *cpr1* loss-of-function mutants, suggesting that the overexpression of these mutated *CPR1* has impact on the normal function of endogenous *CPR1*.

### The MT cytoskeleton is defective during PC morphogenesis in *cpr1* mutants

**Fig 7:**
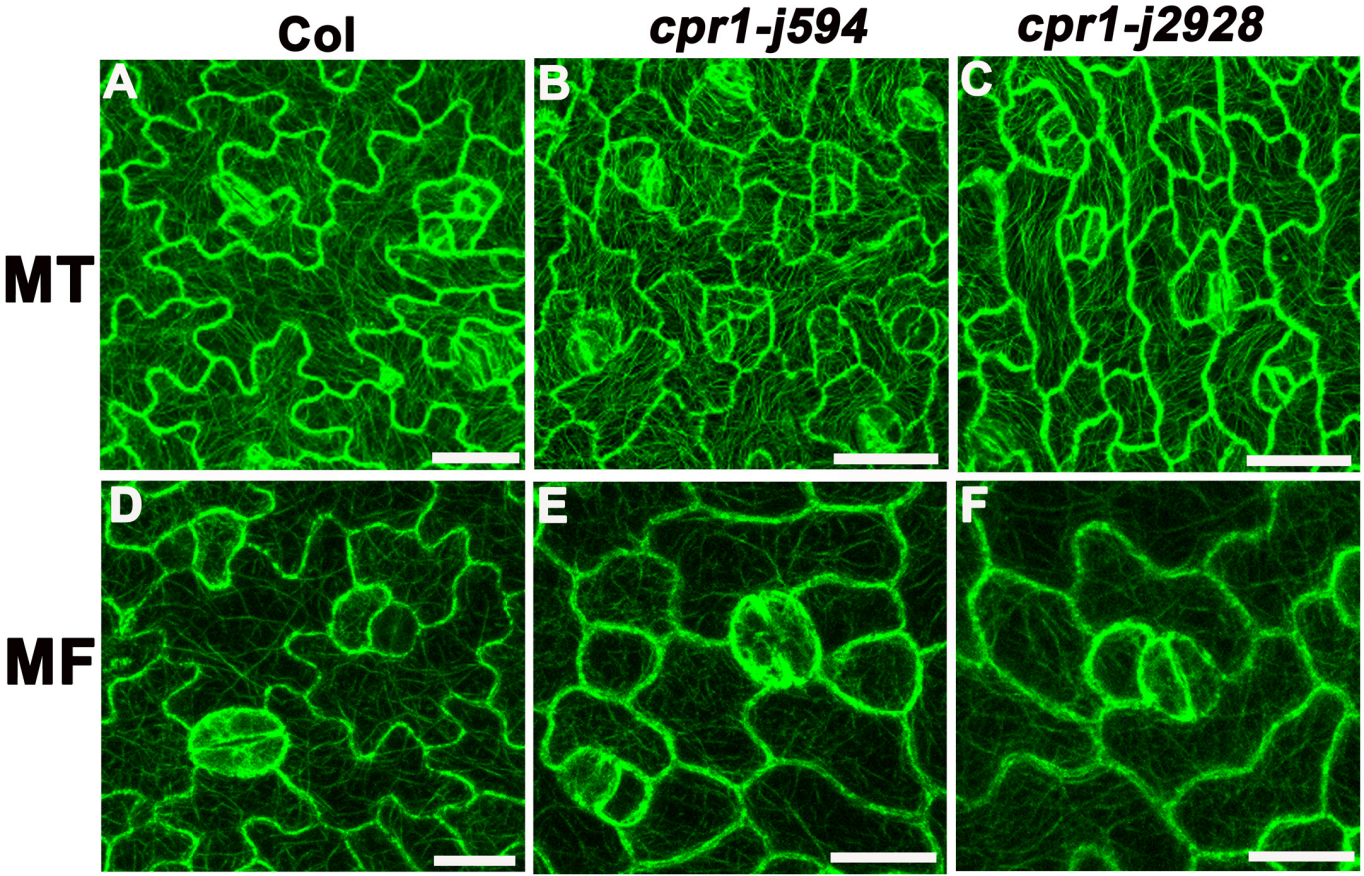
CPR1 is required for both MT and MF ordering. **(A–C)** Cortical microtubule organization and alignment in PCs of Col (**A**), *cpr1-j594* (**B**), and *cpr1-j2928* (**C**). **(D–F)** Microfilaments in PCs from Col (**D**), *cpr1-j594* (**E**), and *cpr1-j2928* (**F**). Bars = 20 μm.

**Fig 8:**
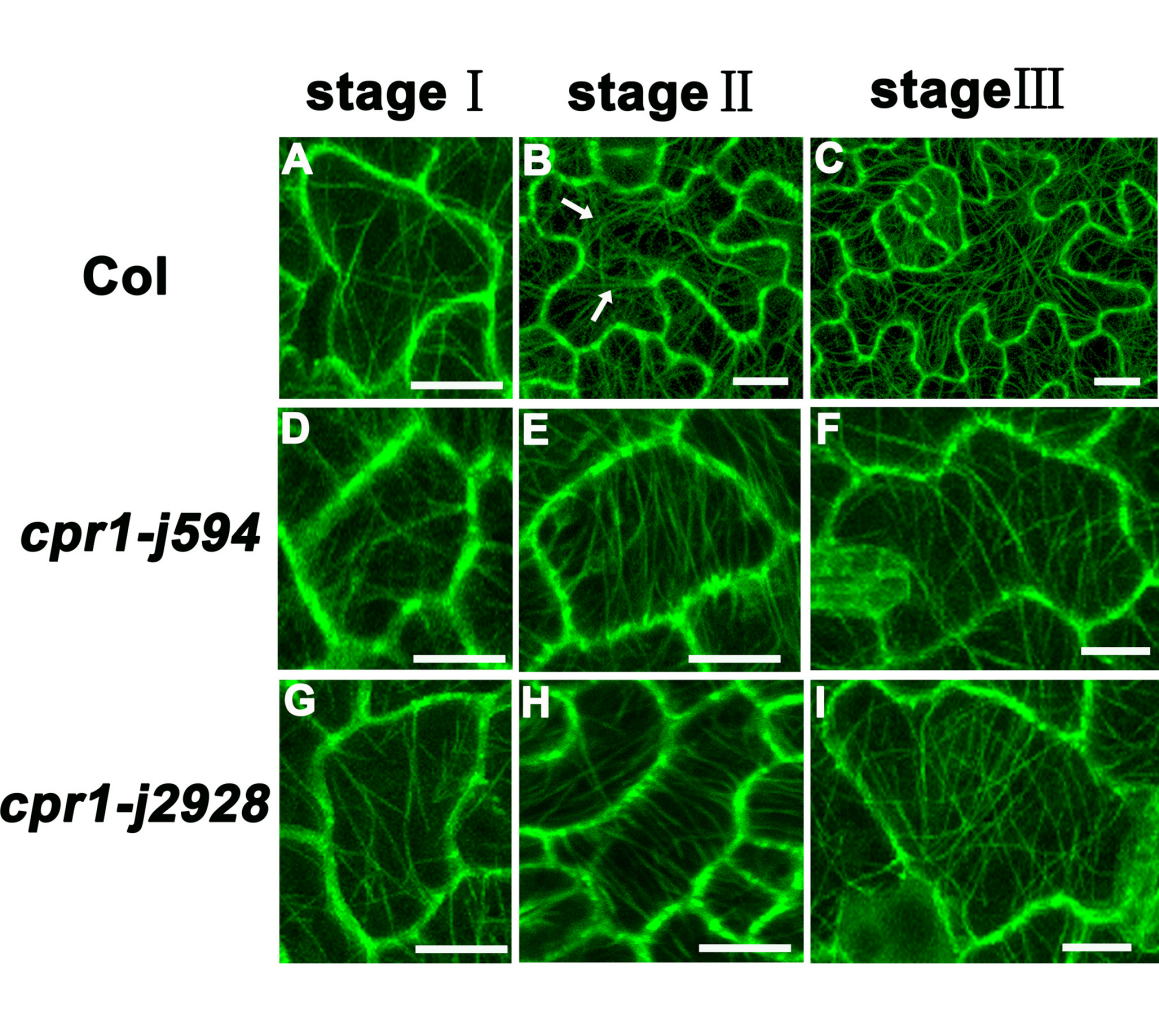
Analysis of MT organization in different development stages of PC. **(A, D, G)** MT organization in stage I PCs from Col (**A**), *cpr1-j594* (**D**), and *cpr1-j2928* (**G**). (**B, E, H)** MT organization in stage II PCs from Col (**B**), c*pr1-j594* (**E**), and *cpr1-j2928* (**H**), the arrows indicated the neck region. **(C, F, I)** MT organization in stage III PCs from Col (**C**), *cpr1-j594* (**F**), and *cpr1-j2928* (**I**). Bars = 20 μm.

Microtubules play an important role in maintaining PC morphogenesis [8,29]. Thus, we wondered whether the microtubule cytoskeleton was changed in *cpr1-j594* and *cpr1-j2928*. The MT cytoskeleton in Col-0 and two *cpr1* mutants was detected using the labeled marker TUA6-GFP. MT arrangement formed simpler cortical MT networks with relatively single oriented arrays in the mature PCs of the two *cpr1* mutants than in wild-type PCs (Fig 7A–7C). To further investigate the differences in MT arrangement among *cpr1-j594*, *cpr1-j2928*, and wild-type PCs during their developmental process, MT arrangement during the three PC developmental stages was visualized. At first, the arrangement of cortical MTs was random in wild-type, *cpr1-j594*, and *cpr1-j2928* PCs (Stage I) (Fig 8A, 8D and 8G). After lobe initiation, transverse MTs were most prominent in the neck region in wild-type PCs (Stage II) (Fig 8B), whereas the MT bundles were arranged in parallel through the whole PCs. (Fig 8E and 8H). Finally, after the full lobe expansion, mature PCs were formed and the cortical MTs exhibited a complex network with randomly oriented arrays in wild-type PCs (Fig 8C). In *cpr1-j*594 and *cpr1-j2928* mature PCs, only simple cortical MT networks were observed (Fig 8F and 8I). All these results suggest that the directional arrangement of MTs during PC development is defective in *cpr1* mutants.

### The arrangement of the actin cytoskeleton is disturbed in *cpr1* mutants

**Fig 9:**
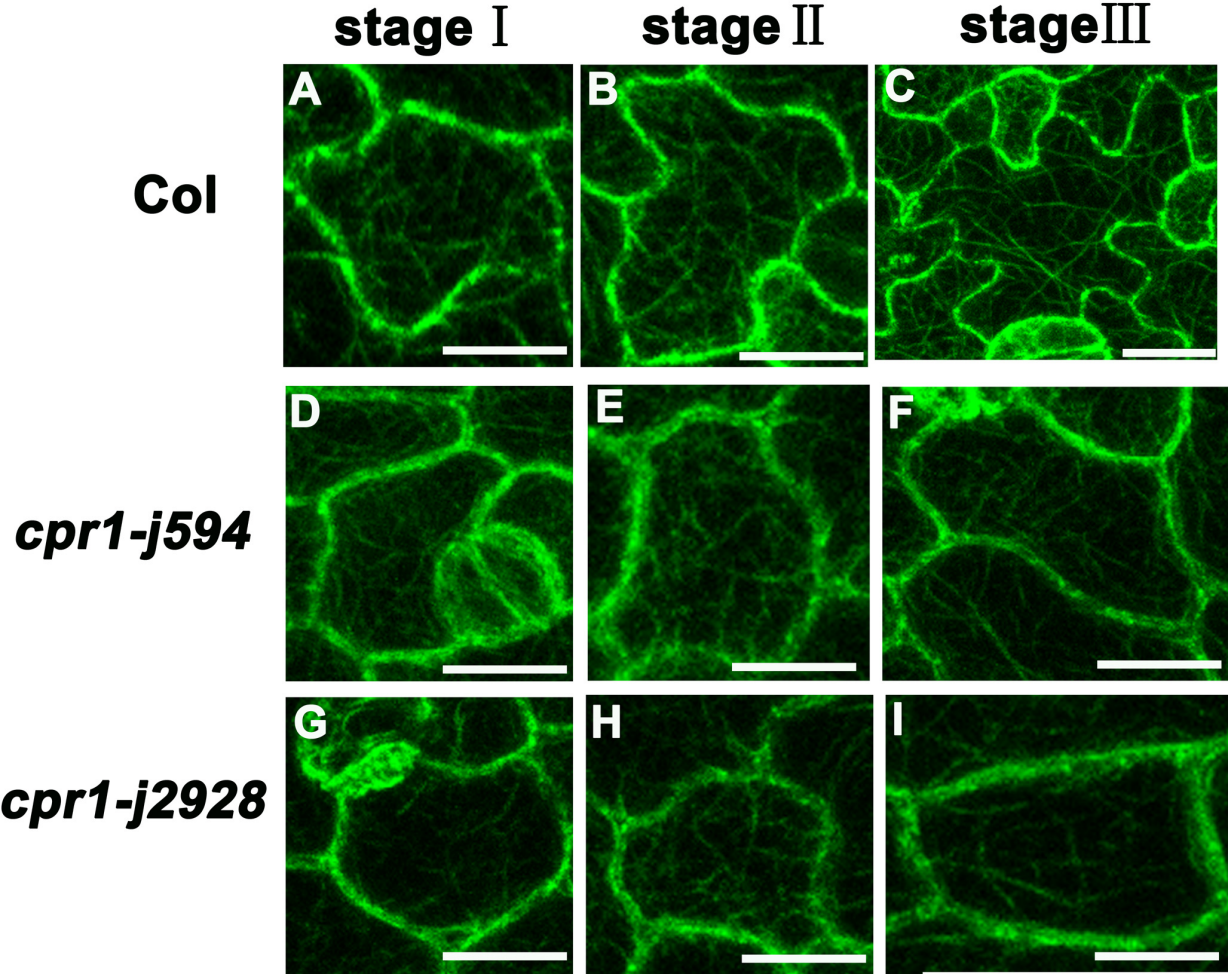
Analysis of MF cytoskeleton in different development stages of PC. **(A**, **D**, **G)** MF cytoskeleton in stage I PCs from Col (**A**), *cpr1-j594* (**D**), and *cpr1-j2928* (**G**). (**B**, **E**, **H)** MF cytoskeleton in stage II PCs from Col (**B**), *cpr1-j594* (**E**), and *cpr1-j2928* (**H**). (**C**, **F**, **I)** MF cytoskeleton in stage III PCs from Col (**C**), *cpr1-j594* (**F**), and *cpr1-j2928* (**I**). Bars = 20 μm.

The actin cytoskeleton is also associated with plant cell polar growth and is essential for proper cell morphogenesis [30]. Therefore, we investigated the actin cytoskeleton in *cpr1*. GFP-FABD2 was used as a molecular marker to label the actin cytoskeleton in the PC. In wild-type PCs, abundant microfilaments formed actin cables networks (Fig 7D), whereas *cpr1-j594* and *cpr1-j2928* PCs did not form obvious networks (Fig 7E and 7F). These results indicated that the distribution and arrangement of the actin cytoskeleton is disturbed in *cpr1*. To determine at which developmental stage during PC morphogenesis started the actin cytoskeleton disruption in *cpr1*, the actin cytoskeleton during the three PC developmental stages was observed. In wild-type PCs, diffuse microfilaments spread throughout the cells at first (Stage I) (Fig 9A). During lobe initiation and expansion, microfilaments began to aggregate in apparently expanding lobes and gradually disappeared in the neck region of the cortex (Stage II). Some thin actin cables were also formed during this stage (Fig 9B). Finally, the cortical diffuse microfilaments disappeared completely, and networks of actin cables were formed (Stage III) (Fig 9C). Little difference between *cpr1-j594*, *cpr1-j2928*, and wild-type PCs was observed during Stages I and II (Fig 9D, 9E, 9G and 9H). However, during the transition from Stage II to III, the cortical diffuse microfilaments in *cpr1-j594* and *cpr1-j2928* PCs did not totally disappeared and did not form apparent MF networks (Fig 9F and 9I). These results suggest that the cortical fine actin filaments cannot be aggregated effectively to form actin cable networks in *cpr1* mutants and this maybe impact on the function of actin cytoskeleton.

### PC morphogenetic defects in *cpr1* require functional *PAD4* and *EDS1*, and are temperature dependent

**Fig 10:**
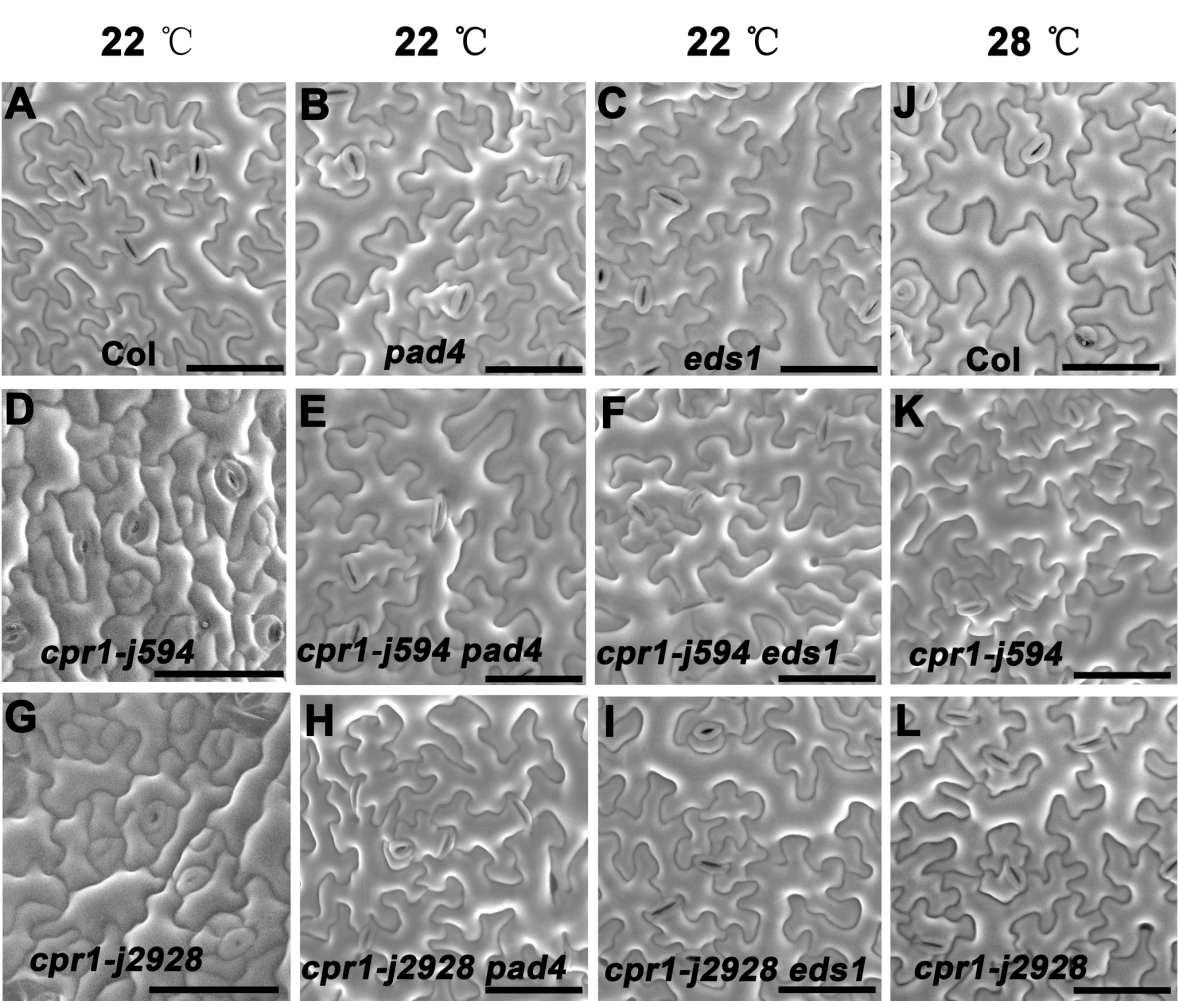
CPR1 regulates PC morphogenesis dependent on natural plant immune response. **(A–I)** Genetic interaction analysis between *CPR1* and *PAD4*, *EDS1* in PC morphogenesis. **(J–L)** Mature PC shape of Col, *cpr1-j594*, *cpr1-j2928* growing at 28°C. Bars = 50 μm.

**Fig 11:**
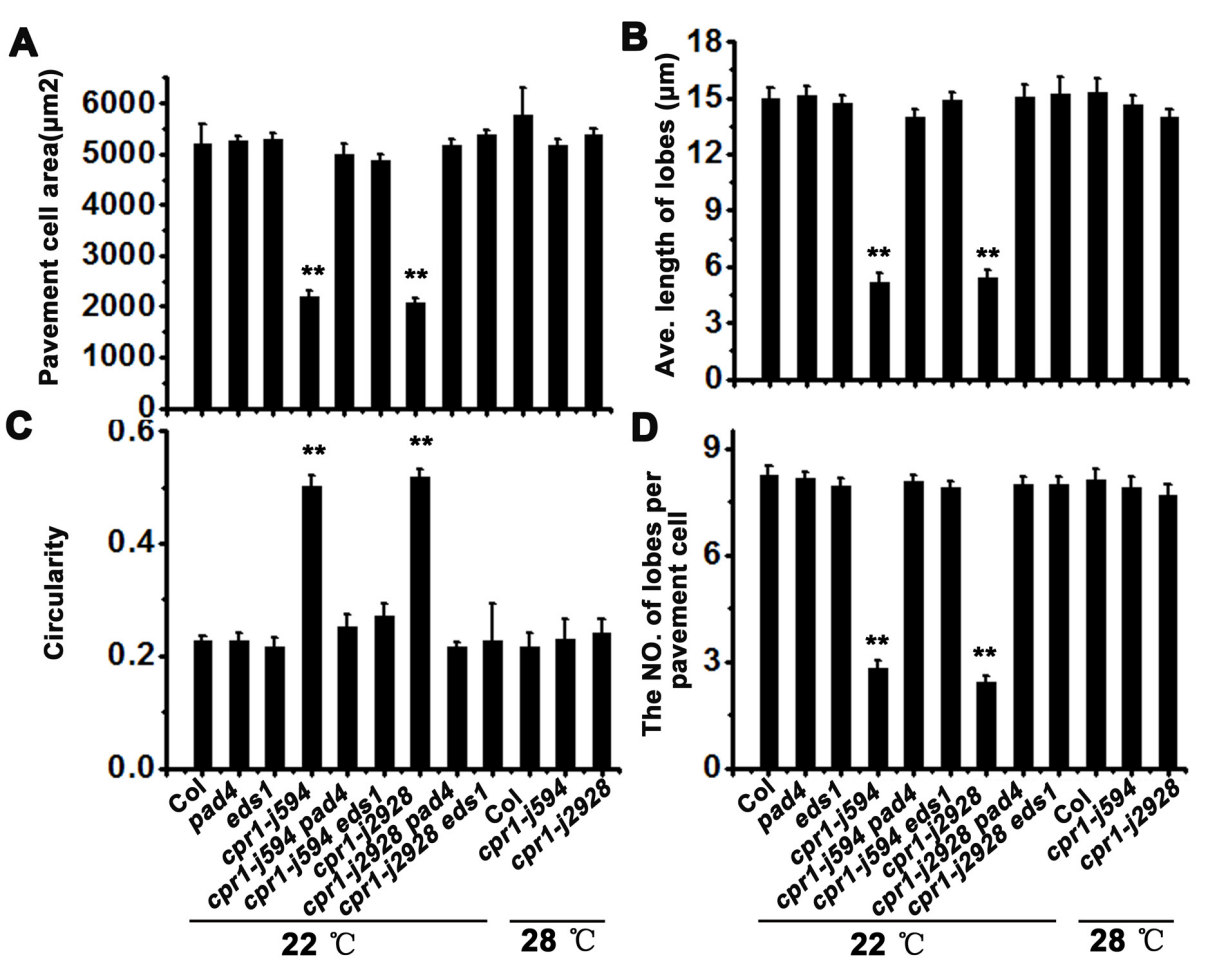
Quantitative analysis of the PC in the high temperature test and the genetic interaction between CPR1 and PAD4, EDS1. PC area (**A**), lobe length (**B**), circularity (**C**), and lobe number (**D**)**.** Error bars indicate s.e.m.; **, P<0.01 by Student’s test.

ROP-GTPase has been reported to be crucial in regulating cytoskeleton organization in PCs [11,29]. However, yeast two hybrid (Y2H) and bimolecular fluorescence complementation (BiFC) assays showed that CPR1 cannot interact with ROP2, ROP4, and ROP6 directly (data not shown). Genetic interaction analysis between CPR1 and ROP2, and CPR1 and ROP6 also showed the CPR1 is not involved in ROP-GTPase signaling pathway (S3 and S4 Figs.)**.** It has been reported that CPR1 negatively regulates defense responses through PAD4 and EDS1, two key components in the plant immune response network [20]. To test whether CPR1 regulates PC morphogenesis through the PAD4/EDS1-mediated signaling pathway, we characterize the PCs in *cpr1 pad4* and *cpr1 eds1* double mutants. PCs in single *pad4* and *eds1* mutants did not exhibited an obvious phenotype (Fig 10B and 10C), but in *cpr1 pad4* and *cpr1 eds1* double mutants the *cpr1*-mediated PC morphogenetic defects were suppressed (Fig 10D–10I; Fig 11). As CPR1-mediated plant immune response is temperature sensitive [25]. We also characterized the PCs of *cpr1* mutants grown at 28°C. The abnormal PC shapes of *cpr1-j594* and *cpr1-j2928* were rescued at 28°C (Fig 10K and 10L; Fig 11), indicating that high temperature can rescue the phenotype of PCs in *cpr1* mutants. Taken together, these results suggest that the CPR1-controlled PC morphogenesis requires normal PAD4 and EDS1, and is temperature dependent.

## Discussion

### The FBA domain is indispensable for normal CPR1 function

The F-box protein is one of the major subunits within the SCF E3 ubiquitin ligase complex which recognizes the substrates for ubiquitination and proteasome-mediated degradation [31]. Many F-box proteins have an F-box-associated (FBA) motif at its C terminus which specifically recognizes the substrates. In our study, overexpression in Col-0 background of *CPR1* with a point mutation in the FBA domain or without the FBA domain exhibited the phenotypes of the CPR1 loss-of-function (Fig 6). These results indicate that the FBA domain is essential for normal CPR1 function and the mutated CPR1 proteins affect the function of endogenous normal CPR1. It has also been reported that the F-box motif of CPR1 interacts with ARABIDOPSIS SKP1 HOMOLOGUE 1 (ASK1) or ARABIDOPSIS SKP1 HOMOLOGUE 2 (ASK2) to form SCF complexes [20]. Based on these results, we speculated that both CPR1 mutant proteins without functional FBA domains, can competitively combine with SKP1 and CULLIN proteins to form SCF complexes, but these complexes fail to recognize theirs substrates for degradation. Due to this competition with the endogenous CPR1, few functional SCF complexes will be formed, reducing the physiological activity and signal transduction in plants. Therefore, *35S*:*CPR1I247V* and *35S*:*CPR1ΔFBA* transgenic plants also showed *cpr1* loss-of-function phenotype.

### CPR1 controls the cytoskeleton arrangement independent of the ROP-GTPase signaling

ROP-GTPases work as a key switcher that mediate the interactions between the actin and microtubule cytoskeletons, controlling the high interdigitation of PCs [8,10,11,29,32]. In animal cells, F-box/LRR-repeat protein 19 (FBXL19) controls cell morphogenesis through the ubiquitination and degradation of small GTPase proteins including RhoA (RAS HOMOLOG FAMILY MEMBER A), RAC1 (RAS-RELATED C3 BOTULINUM TOXIN SUBSTRATE 1), and RAC3 [33–35], which are involved in polar cell growth and morphogenesis [36]. However, how F-box proteins influence the polar growth and morphogenesis of PCs in *Arabidopsis* is still unknown. ROP6, a small Rho GTPase involved in plant pathogen responses [37], exhibits similar PC morphogenesis phenotype to that of *cpr1* when it is constitutively activated in plants [10], implying that CPR1 might take part in the degradation of the small Rho GTPases. However, both *cpr1 rop6*, *cpr1 rop2* double mutants and *cpr1rop6 rop2* triple mutant cannot rescue the phenotype of *cpr1* (S3 and S4 Figs.). Protein interaction assays also proved that CPR1 cannot interact with ROP2, ROP4, and ROP6. Therefore, ROP-GTPases may not be CPR1 substrates. Comparison of the amino acid sequences of CPR1 and FBXL19 showed that both have an F-box domain within the N-terminus; however, FBXL19 possesses a unique leucine-rich repeat in the C-terminus, which has been proved to interact with RAC3 in animal cells [33]. Based on this analysis, we speculated that some F-box proteins with the unique leucine-rich repeat in the C-terminus domain may play a role in the ubiquitin-degradation of the small Rho GTPase during PC morphogenesis.

### CPR1-mediated control of the cytoskeleton arrangement depends, at least in part, on the plant defense responses

The cytoskeleton plays an active role in modulating the response to various types of antimicrobial defenses by blocking fungal pathogen penetration of the surrounding cells [38–40]. Studies on simple and complex cells, such as cylindrical hypocotyl cells and leaf epidermal PCs, respectively, have produced a mass of evidence to prove the importance of the cytoskeleton in control of cell shape [8,11,15]. In our study, the high temperature and the loss-of-function of *PAD4* and *EDS1* can completely rescue the PC morphogenetic defects in *cpr1* mutants (Fig 10). In addition, MT arrangement was also rescued and exhibited a complex network with randomly oriented arrays in *cpr1* mutants grown at 28°C (S5 Fig), further indicating that the cytoskeleton defects in the PCs of *cpr1* are temperature dependent. Temperature modulates plant defense responses through NB-LRR proteins, which are positively regulated by EDS1 and PAD4 [25]. It has also been reported that EDS1 and PAD4 have a higher steady expression level at 22°C than at 28°C [41]. Thus, high temperature and, the loss-of-function of *PAD4* and *EDS1* could both rescue the morphogenetic defects in the PCs of *cpr1* mutants due to the inhibition to the PAD4/EDS1-mediated defense response. Taken together, these results suggest that the CPR1-mediated control of the cytoskeleton arrangement depends, at least in part, on the plant defense responses.

## Conclusions

Our study demonstrates that CPR1-regulated cytoskeleton arrangement controls pavement cell morphogenesis, which is involved in plant defense responses and that the FBA domain is essential for normal CPR1 function.

## Supporting Information

S1 Fig Relative expression of *CPR1* in Col, *35S*::*CPR1*, *35S*::*CPR1I247V* and *35S*::*CPR1∆FBA* transgenic plants. (P1+P2) represents endogenous transcription level of *CPR1*, (P3+P4) represents total transcription level of *CPR1*.
(TIF)

S2 Fig Quantitative analysis of the stage I and stage II PCs in Col, *j594*, *j2928* and SALK_045148. lobe number (A), PC perimeter (B) and PC area (C).
(TIF)

S3 Fig Genetic interaction analysis between CPR1 and ROP6/ROP2. **(A)**Two-week-old seedlings of Col, *rop6*, *rop2*, *rop6 rop2*, *cpr1-j594*, *cpr1-j2928*, *cpr1-j594 rop6*, *cpr1-j594 rop2*, *cpr1-j594 rop6 rop2*, *cpr1-j2928 rop6*, *cpr1-j2928 rop2*, and *cpr1-j2928 rop6 rop2*. Bars = 1 cm. **(B)** Mature PC shape of Col, *rop6*, *rop2*, *rop6 rop2*, *cpr1-j594*, *cpr1-j2928*, *cpr1-j594 rop6*, *cpr1-j594 rop2*, *cpr1-j594 rop6 rop2*, *cpr1-j2928 rop6*, *cpr1-j2928 rop2*, and *cpr1-j2928 rop6 rop2*. Bars = 50 μm.
(TIF)

S4 Fig Quantitative analysis of the PCs in the genetic interaction between CPR1 and ROP6/ROP2. PC area (**A**), lobe length (**B**), circularity (**C**), and lobe number (**D**)**.**
(TIF)

S5 Fig The MT arrangement of *cpr1-j594* and *cpr1-j2928* can be rescued at 28°C. **(A)** and (B) Cortical microtubule organization and alignment in PCs of *cpr1-j594* (A), and *cpr1-j2928* (B) growing at 22°C. (C) and (D) Cortical microtubule organization and alignment in PCs of *cpr1-j594* (C), and *cpr1-j2928* (D) growing at 28°C. Bars = 50 μm.
(TIF)

S1 Table Primers used in this study. (DOCX)
