# Supplementary figures and images for: Constitutive Expresser of Pathogenesis Related Genes 1 Is Required for Pavement Cell Morphogenesis in Arabidopsis
Source: PLoS One. 2015 Jul 20;10(7):e0133249. doi: 10.1371/journal.pone.0133249 (PMC4508093; doi:10.1371/journal.pone.0133249)

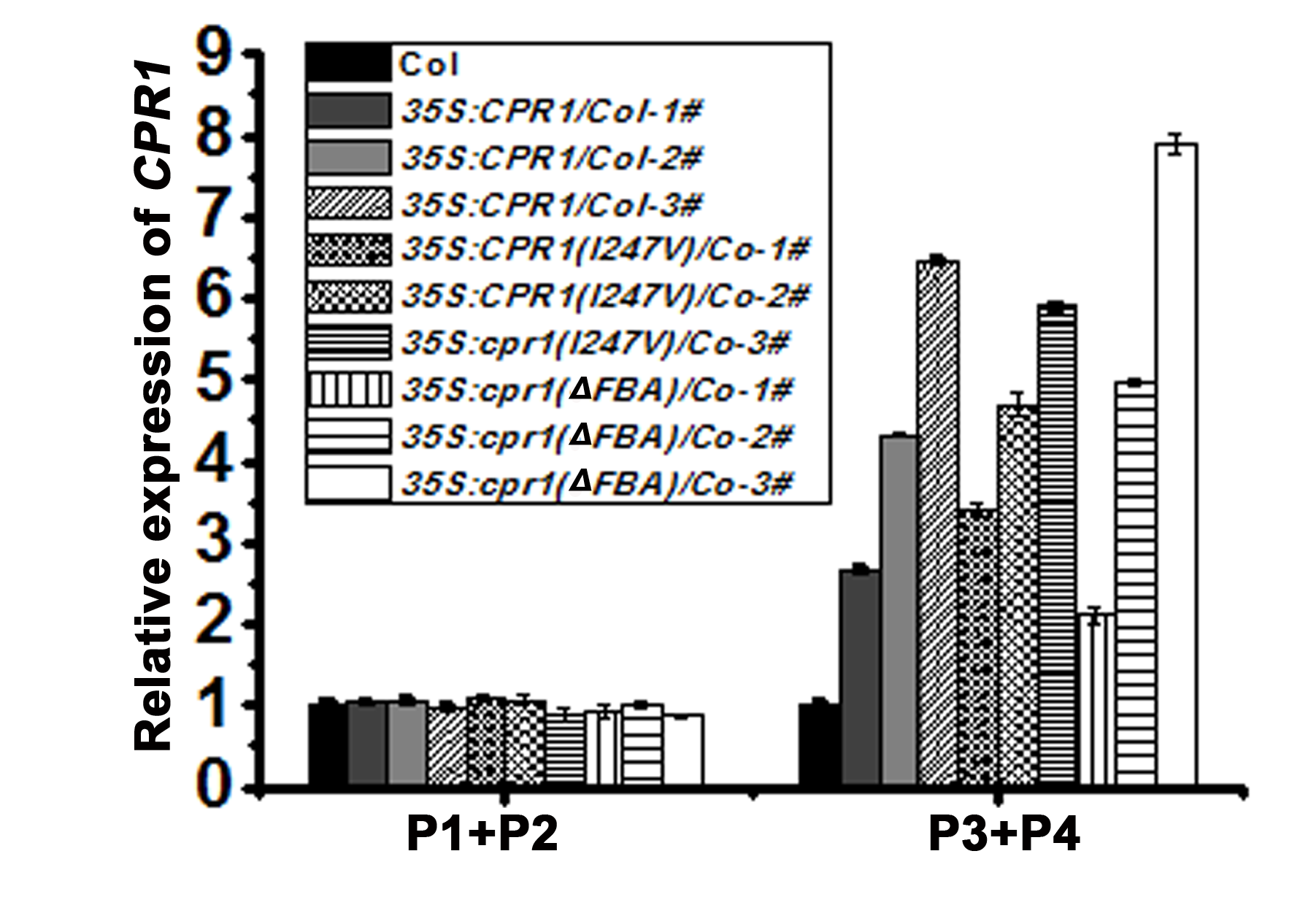

Supplement: S1 Fig — (P1+P2) represents endogenous transcription level of CPR1, (P3+P4) represents total transcription level of CPR1. (TIF) [file pone.0133249.s001.tif]

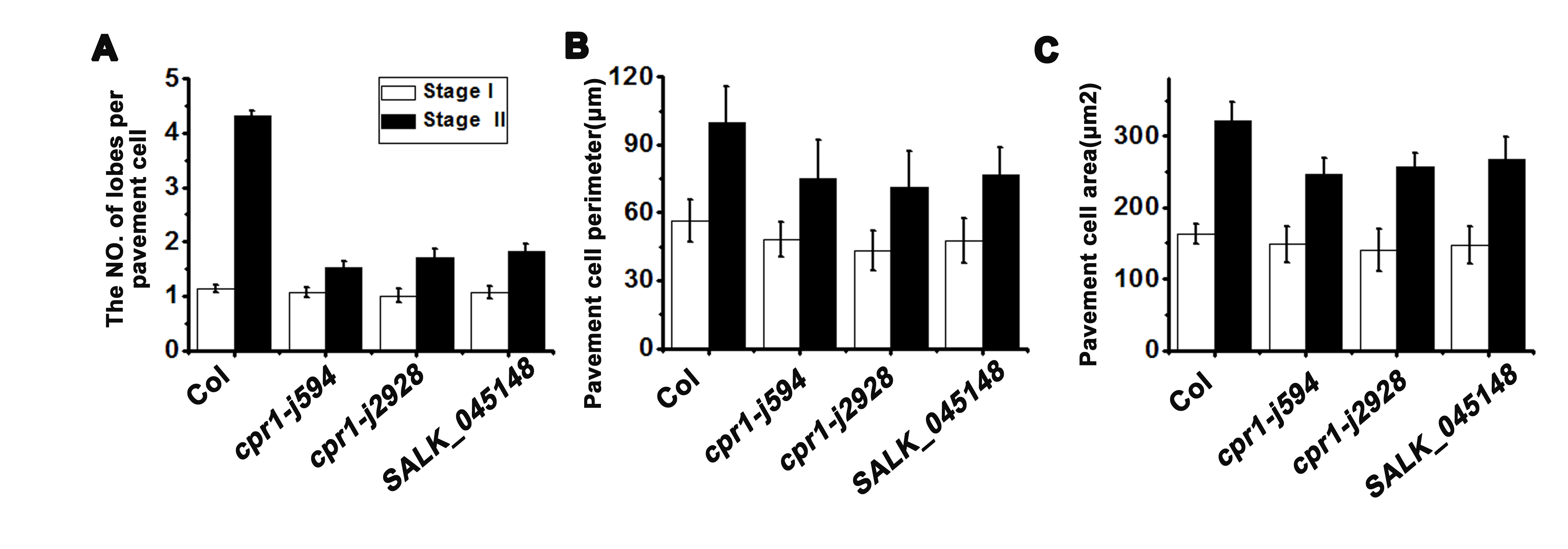

Supplement: S2 Fig — lobe number (A), PC perimeter (B) and PC area (C). (TIF) [file pone.0133249.s002.tif]

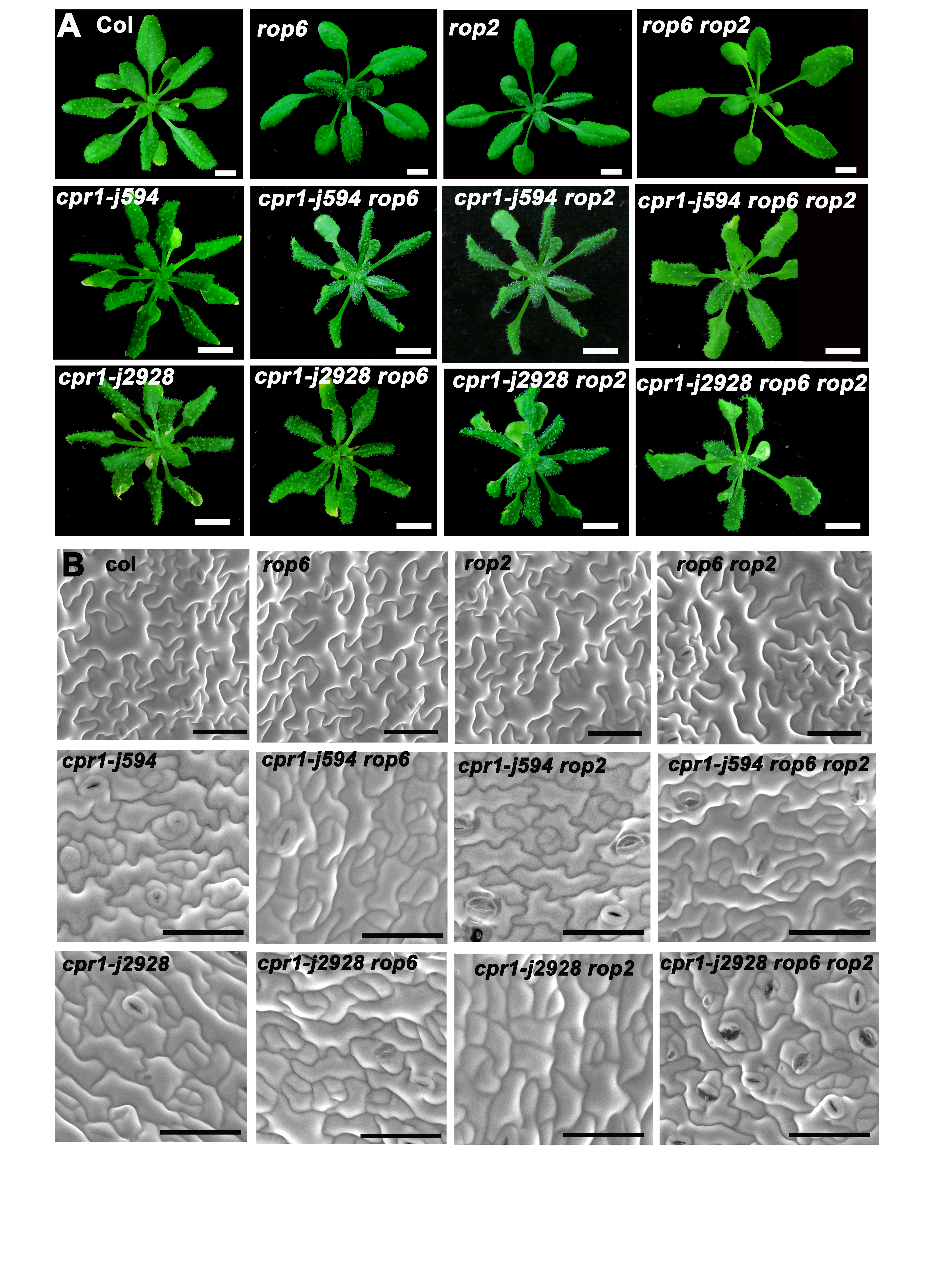

Supplement: S3 Fig — (A)Two-week-old seedlings of Col, rop6, rop2, rop6 rop2, cpr1-j594, cpr1-j2928, cpr1-j594 rop6, cpr1-j594 rop2, cpr1-j594 rop6 rop2, cpr1-j2928 rop6, cpr1-j2928 rop2, and cpr1-j2928 rop6 rop2. Bars = 1 cm. (B) Mature PC shape of Col, rop6, rop2, rop6 rop2, cpr1-j594, cpr1-j2928, cpr1-j594 rop6, cpr1-j594 rop2, cpr1-j594 rop6 rop2, cpr1-j2928 rop6, cpr1-j2928 rop2, and cpr1-j2928 rop6 rop2. Bars = 50 μm. (TIF) [file pone.0133249.s003.tif]

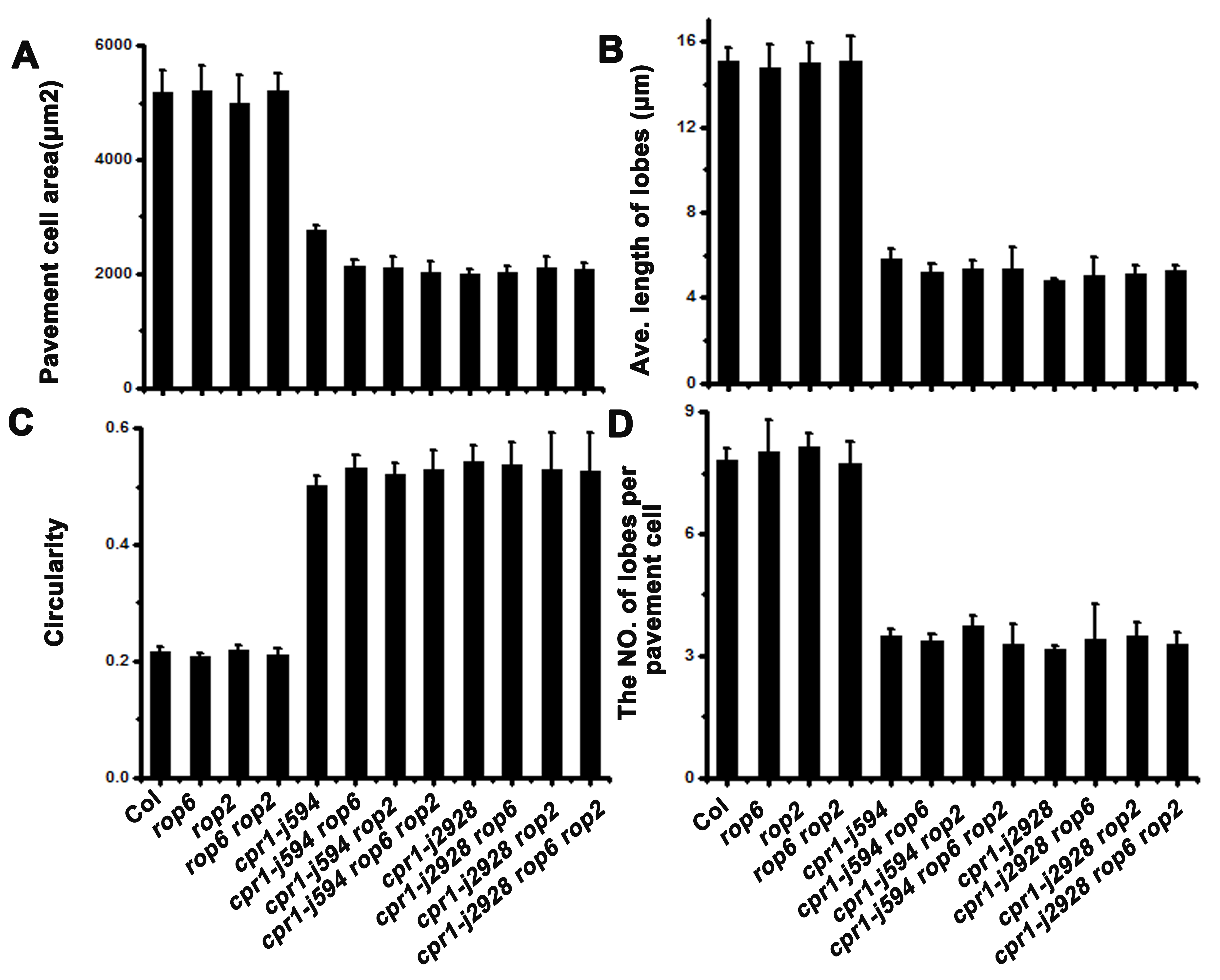

Supplement: S4 Fig — PC area (A), lobe length (B), circularity (C), and lobe number (D). (TIF) [file pone.0133249.s004.tif]

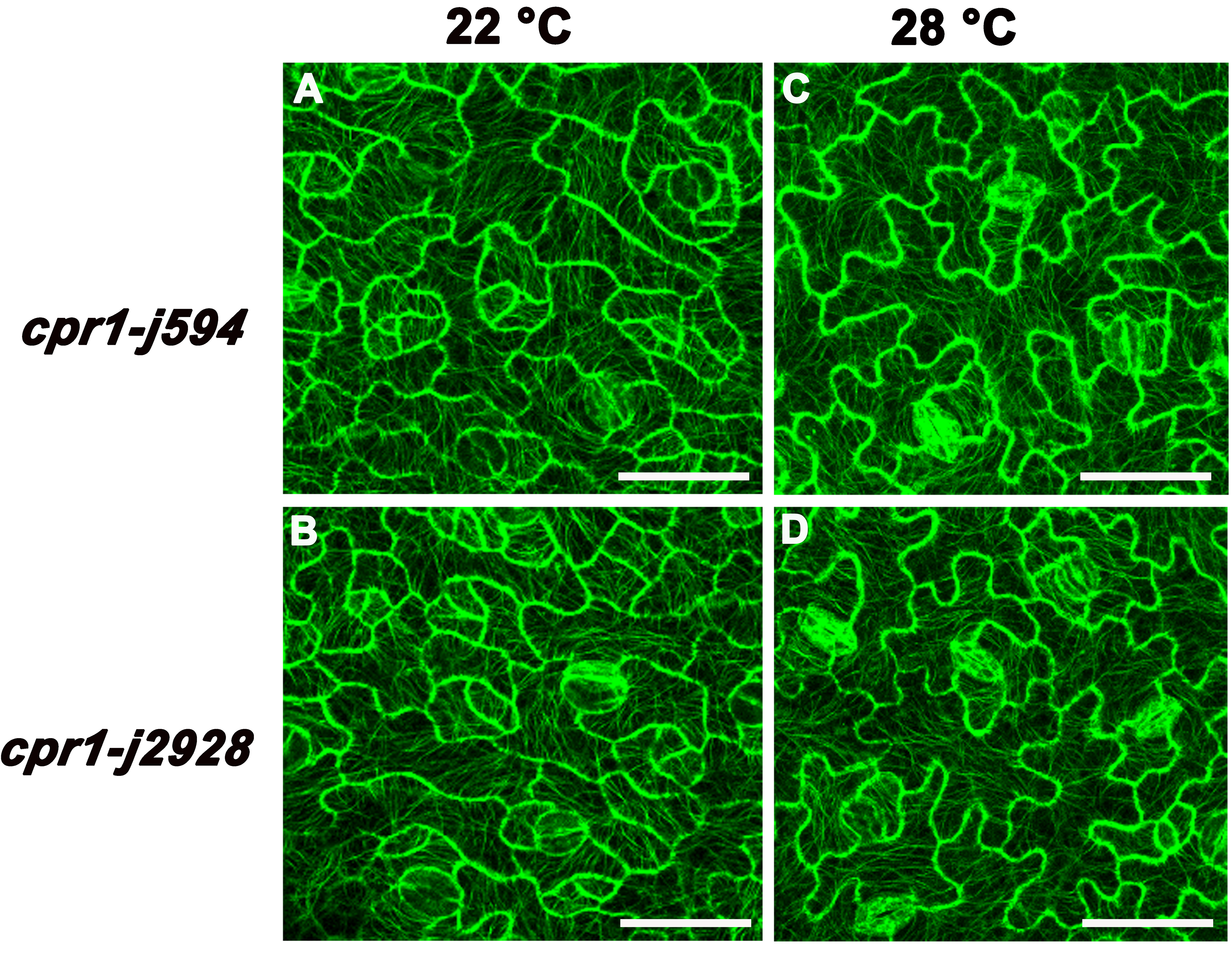

Supplement: S5 Fig — (A) and (B) Cortical microtubule organization and alignment in PCs of cpr1-j594 (A), and cpr1-j2928 (B) growing at 22°C. (C) and (D) Cortical microtubule organization and alignment in PCs of cpr1-j594 (C), and cpr1-j2928 (D) growing at 28°C. Bars = 50 μm. (TIF) [file pone.0133249.s005.tif]
