# Supplementary material for: Constitutive Expresser of Pathogenesis Related Genes 1 Is Required for Pavement Cell Morphogenesis in Arabidopsis
Source: PLoS One. 2015 Jul 20;10(7):e0133249. doi: 10.1371/journal.pone.0133249 (PMC4508093; doi:10.1371/journal.pone.0133249)
Supplement: S1 Table — (DOCX) [file pone.0133249.s006.docx]

**S1 Table.** Primers Used in This Study.

| **Primer name** | **Sequence** | |
| --- | --- | --- |
| **Complementation test** | | |
| *CPR1-*PB | AAAAAAGCAGGCTTCcctcaactccactaggtgc | |
| *CPR1-*PB2 | CAAGAAAGCTGGGTTCTTGTGCAGCTTTAGCTCC | |
| **Genotyping** | | |
| *SALK_045148-R*  *SALK_045148-L* | | TgTgAgTAgCCTTgTCTTggg  TTTCgTAAATTTTTACACAAAATCg |
| *SALK_LBa1* | | TGGTTCACGTAGTGGGCCATCG |
| **Overexpression** | | |
| 35S:CPR1-F | AAAAAAGCAGGCTTCATGGCGACGATTCCAATGG | |
| 35S:CPR1-R  35S:∆FBA-P1  35S:∆FBA-P2 | CAAGAAAGCTGGGTTTAAGACCAGCTTGAATCCT  CCTAGAAGACCTGGTCTTGTCTTGGGGTGTAAAG  TACACCCCAAGACAAGACCAGGTCTTCTAGGTAA | |
| **RT-PCR** | | |
| P1 | CGACGATTCCAATGGATATCG | |
| P2 | GAAGAACGCGATGAAGATGAG | |
| P3 | GGAACTCGTTGTGAGTAGCC | |
| P4 | GTGCAGCTTTAGCTCCTGTC | |
| **Yeast two hybridization** | | |
| ROP2AD-F | CATATGATGGCGTCAAGGTTTATAAAG | |
| ROP2AD-R | GAATTCCAAGAACGCGCAACGGTTCTT | |
| ROP4AD-F | CATATGATGAGTGCTTCGAGGTTT | |
| ROP4AD-R | GAATTCCAAGAACACGCAGCGGTTC | |
| ROP6AD-F | CATATGATGAGTGCTTCAAGGTTT | |
| ROP2AD-R  CPR1BD-F  CPR1BD-R | GAATTCGAGTATAGAACAACCTTTC  CAT ATG ATGGCGACGATTCCAATGGATATCGTCAACG  CGG ATC CTA TAAGACCAGCTTGAATCCTTTGGACAGG | |
| **BiFC** | | |
| ROP2-YN-F | AAAAAAGCAGGCTTCATGGCGTCAAGGTTTATAAAG | |
| ROP2-YN-R | CAAGAAAGCTGGGTTCAAGAACGCGCAACGGTTC | |
| ROP4-YN-F | AAAAAAGCAGGCTTCATGAGTGCTTCGAGGTTTA | |
| ROP4-YN-R | CAAGAAAGCTGGGTTCAAGAACACGCAGCGGTTC | |
| ROP6-YN-F | AAAAAAGCAGGCTTCATGAGTGCTTCAAGGTTTATC | |
| ROP6-YN-R  CPR1-YC-F  CPR1-YC-R | CAAGAAAGCTGGGTTGAGTATAGAACAACCTTTCTG GGGGACAAGTTTGTACAAAAAAGCAGGCTTCATGGCGACGATTCCAATGG  GGGGACCACTTTGTACAAGAAAGCTGGGTTTAAGACCAGCTTGAATCCT | |
